# Anti-Cell Staining Patterns in Juvenile Idiopathic Arthritis-Associated Uveitis: A Peek Behind the Curtain

**DOI:** 10.3390/jcm15103977

**Published:** 2026-05-21

**Authors:** Marijan Frkovic, Ivana Sabljak, Nada Tomic Sremec, Domagoj Kifer, Ana Kozmar, Sanja Peric, Marija Barisic Kutija, Iva Rukavina, Mario Sestan, Nastasia Kifer, Sanda Huljev Frkovic, Marija Jelusic

**Affiliations:** 1 Department of Paediatrics, University Hospital Centre Zagreb, 10000 Zagreb, Croatia; mfrkovic1@gmail.com (M.F.);; 2 School of Medicine, University of Zagreb, 10000 Zagreb, Croatia; 3 Department of Laboratory Diagnostics, University Hospital Centre Zagreb, 10000 Zagreb, Croatia; 4 Faculty of Pharmacy and Biochemistry, University of Zagreb, 10000 Zagreb, Croatia; 5 Department of Ophthalmology, University Hospital Centre Zagreb, 10000 Zagreb, Croatia

**Keywords:** juvenile idiopathic arthritis, uveitis, antinuclear antibodies, staining patterns, titres

## Abstract

**Objectives:** We aimed to assess and compare Anti-Cell (AC) staining patterns and semi-quantitative titre levels of related antibodies in patients with juvenile idiopathic arthritis (JIA) with and without associated uveitis (JIA-U) to identify distinctive immunological profiles and contribute to a deeper understanding of JIA-U aetiopathogenesis. **Methods**: Data from JIA patients diagnosed between January 2020 and December 2025 in our centre were evaluated. The HEp-2 indirect immunofluorescence assay (HEp-2 IFA) was used to determine AC patterns and semiquantitative titres of associated antibodies. **Results:** 217 JIA patients were evaluated: 161 (74%) without uveitis and 56 (26%) with JIA-U. The AC-1 pattern was detected in 94 of 217 children (43%). It was markedly enriched among children with JIA-U, present in 39 of 56 (70%), compared with 55 of 161 (34%) in the non-uveitis group. High staining intensity (i.e., semiquantitative titre ++/+++) of AC-1 was also more commonly detected in JIA-U patients (39%) compared to children without uveitis (11.8%). The AC-4 pattern was detected in 30 of 217 JIA patients (14%), but more commonly in the non-uveitis (17.4%) compared to the JIA-U group (3.6%). **Conclusions:** JIA-U is closely linked to AC-1 staining patterns and higher semiquantitative titres of associated antibodies. Our results provide additional insight into the pathogenesis of JIA-U and underscore the significance of the new Paediatric Rheumatology International Trials Organisation (PRINTO) JIA classification criteria. The potential clinical relevance of AC patterns in children with JIA and JIA-U requires further multicentre studies involving larger cohorts and extended follow-up periods.

## 1. Introduction

Juvenile idiopathic arthritis (JIA) is the most common rheumatic disease in childhood [1,2]. It encompasses a group of chronic arthritis with incompletely elucidated aetiology and onset before the age of 16 [1,2,3]. The International League Against Rheumatism (ILAR) classifies JIA into seven mutually exclusive categories based on joint count, extra-articular features, and laboratory markers, including rheumatoid factor (RF) and human leucocyte antigen (HLA) B27 [4]. The disease is more common in girls than in boys (ratio 2:1), with varieties in distribution across all categories and biphasic peaks in age at onset in oligoarticular (oJIA), polyarticular rheumatoid factor negative (pJIA RF-), and psoriatic types of the disease (PsJIA) [1,2].

Decades of data collection and subsequent advancements in the understanding of JIA pathophysiology, treatments, and outcomes exposed shortcomings in the ILAR classification, primarily the lack of a connection to pathogenesis, molecular pathways, and therapeutic response [1,2,3,5,6,7,8]. The new proposed Paediatric Rheumatology International Trials Organisation (PRINTO) classification of JIA from 2019, primarily based on aetiopathogenesis, aims to capture childhood counterparts of adult disease, alongside the antinuclear antibody (ANA) positive, early-onset form unique to children under the age of 6 [5,6,7]. Defining the specific form in children, which accounts for up to 50% of all JIA cases, relies on the shared phenotype observed in patients with oJIA, pJIA RF-, and PsJIA, namely female predominance, asymmetric arthritis, a higher risk of uveitis (JIA-U), and, more recently, HLA-DR8/DRB1 positivity and identical B-cell signatures [1,2,3,4,5,6,8,9,10]. However, the new evidence-based classification must be validated in large cohorts of new JIA patients, and the final set of inclusion criteria and nomenclature, with the possibility of adding new genetic and/or biologic types, are still to be discussed and defined by experts [6,7,8,10]. Current JIA treatment guidelines include non-steroidal anti-inflammatory drugs (NSAIDs), intra-articular glucocorticoid injections, and disease-modifying anti-rheumatic drugs (DMARDs) [1,2,9,11,12].

The Standardisation of Uveitis Nomenclature (SUN) working group defines uveitis classification based on anatomical location and disease duration [13]. JIA-U is the most frequent extra-articular manifestation of the disease, with an overall prevalence ranging from 9% to 30% [12,14,15,16,17,18,19]. Chronic anterior uveitis accounts for ≥90% of all JIA-U and is most detected in the ANA-positive, early-onset group of JIA patients [3,7,12,14,15,17,18,19,20,21]. The aetiopathogenesis of JIA-U is rather complex and diverse, associated with several HLA genes, a high level of B-cell involvement, and local antibody production that is highly dependable on the duration and disease stages of JIA [14,16,17,18,21,22,23,24,25,26,27]. In addition to ANA, a wide array of autoantibodies primarily targeting iris antigens, such as histones and chromatin, S arrestin (S antigen), retinol binding protein 3 (RBP3), tyrosinase related proteins, heat shock cognate 71 kDa, and keratin, are presumed to contribute to the autoimmune process [14,15,16,18,24,25,28,29]. The onset of chronic JIA-U is often insidious and initially completely asymptomatic (in contrast to the acute, symptomatic onset of anterior uveitis that can be seen in the enthesitis-related form of JIA), and it can affect one or both eyes [14,15,16]. It is diagnosed by slit-lamp examination, usually at the time or within several years after the JIA diagnosis, whereas in <10% of patients, it occurs before the onset of arthritis [14,15,18]. JIA-U is the most serious extra-articular complication of JIA and can result in sight-threatening ocular complications, including cataract, synechiae, band keratopathy, and glaucoma, affecting 15–50% of patients [14,15,17,18,19]. The management of JIA-U involves the use of topical and systemic medications according to recent recommendations, but without uniform recommendations related to the optimal timing and duration of therapy, as well as a high proportion of relapses after therapy withdrawal [14,15,30,31,32]. DMARDs employed for treating JIA reduce the risk of JIA-U onset, particularly when introduced early and in patients with a higher risk of JIA-U development [11,12,14,15,33,34].

According to the new PRINTO JIA classification, one of the inclusion criteria for the ANA-positive, early-onset JIA type is the presence of two positive results of ANA test with a titre ≥ 160, conducted at least 3 months apart [6,35].

ANA positivity is detected in up to 18% of healthy children [6,20,22,29,35,36,37]. Among all JIA patients, the overall prevalence is between 30 and 50% [20,29,36,38], whereas among JIA-U patients, the prevalence varies between 14 and 98% [20,29,36,38,39,40]. It does not increase the risk of JIA, but it increases the risk of chronic JIA-U and other comorbid autoimmune diseases within the first 7 years after JIA onset [3,8,9,20,22,29,35,36,38]. Several studies have shown that a single positive ANA test is sufficient to impart a risk of chronic JIA-U, regardless of the titre [6,8,20,22]. Since ANA production in JIA patients is strongly associated with human leukocyte antigen (HLA) DRB1*08, DRB1*1, and DPB1*02, it is generally considered to be a genetically driven phenomenon (closely related to the ANA-positive, early-onset JIA type). However, the pathogenic role of ANA in JIA-U remains unclear, as does the impact of systemic therapy, particularly TNFα inhibitors, on ANA test results [3,6,8,16,20,22,28,36,38].

The International Consensus on ANA Patterns (ICAP) nomenclature is a standardised, consensus-based classification system for reporting the results of the indirect immunofluorescence assay on HEp-2 cells, with recent publications favouring the term HEp-2 IFA over the historically known ANA test [35,37,41,42,43,44]. It assigns an anti-cellular (AC) code, ranging from AC-0 (negative) to AC-31, to nuclear, cytoplasmic, and mitotic patterns, providing details on prior nomenclature, microscopic findings, and antigenic associations, thereby enhancing consistency in the reporting and clinical interpretation of various autoimmune diseases [35,41,42,44]. Given that most publications on JIA fail to mention AC patterns, and only a few recent papers indicate the possibility of an increased frequency of specific AC patterns in patients with JIA or JIA-U, a well-defined set of AC patterns has yet to be specified for the classification and prognosis of early-onset JIA and associated increased risk of JIA-U [1,10,20,22,26,28,35,45].

This study aimed to determine the association between particular AC patterns and the occurrence of JIA-U to identify distinct immunological profiles and enhance our understanding of the aetiopathogenesis of JIA and JIA-U.

## 2. Materials and Methods

This retrospective/prospective study was conducted at the Department of Paediatrics, Department of Laboratory Diagnostics, and Department of Ophthalmology, University Hospital Centre Zagreb, Croatia, with the approval of the Ethics Committee of the University Hospital Centre Zagreb (No 02/013 AG, Class 8.1-24/76-3, 28 March 2024). It was performed according to the ethical standards of the 1964 Declaration of Helsinki. Parental/legal guardian written consent was obtained for all patients.

### 2.1. Patients and Data

We assessed all JIA patients diagnosed and regularly followed between January 2020 and December 2025. We conducted a retrospective evaluation of electronic medical records for patients diagnosed between January 2020 and March 2024 and followed them prospectively until the end of 2025. Additionally, we enrolled newly diagnosed patients from March 2024 through to the end of 2025 and followed them until the study’s conclusion.

All JIA patients were diagnosed according to the ILAR classification criteria by subspecialists in paediatric rheumatology [4]. JIA-U diagnosis and management were performed by a specialised ophthalmology team following the standardisation of uveitis nomenclature (SUN) working group guidelines [13].

Demographic and clinical features, including JIA subtypes, diagnosis of uveitis, laboratory findings, and applied therapy, were recorded.

This study excluded patients with JIA who were referred to our department from other institutions for a second opinion on diagnosis or treatment, patients with concomitant immunodeficiencies, systemic autoimmune or autoinflammatory conditions, and patients with uveitis caused by factors other than JIA-U.

### 2.2. Analysis of Routine Laboratory Findings

A complete blood count (CBC) was performed using EDTA-anticoagulated venous blood samples on an automated haematology analyser (Sysmex XN-3000, Sysmex Corporation, Kobe, Japan). This system utilises fluorescence flow cytometry for white blood cell differentiation, impedance measurement with hydrodynamics focusing for erythrocyte and platelet counts, and a non-cyanide sodium lauryl sulphate method for haemoglobin determination. Erythrocyte sedimentation rate (ESR) was measured from the same primary tube using an automated analyser (CUBE 30 Touch, Diesse Diagnostica Senese S.p.A., Siena, Italy) and a modified Westergren method. Serum biochemical analyses were performed on an automated clinical chemistry analyser (Alinity ci, Abbott Laboratories, Abbott Park, IL, USA). Serum iron was determined using a direct colorimetric method, in which reduced iron reacts with ferene to form a coloured complex, with intensity proportional to iron concentration. Ferritin levels were measured using a chemiluminescent microparticle immunoassay (CMIA) based on a sandwich immunoassay principle where ferritin binds to anti-ferritin-coated microparticles and an acridinium-labelled conjugate. C-reactive protein (CRP), immunoglobulin G (IgG), immunoglobulin M (IgM), and immunoglobulin A (IgA) concentrations were measured using immunoturbidimetric assays. Serum protein electrophoresis was performed by capillary electrophoresis in silica capillaries under high-voltage conditions using an alkaline buffer system (Capillaris 3 Octa, Sebia, Lisses, France).

### 2.3. Analysis of HEp-2 IFA Results-AC Patterns

Since January 2020, HEp-2 IFA has been routinely/systematically used in our Department of Laboratory Diagnostics to determine AC patterns and semiquantitative titres in all newly diagnosed JIA patients.

The presence of ANA was determined in the sera using HEp-2 IFA in standard 1:100 dilutions (EUROIMMUN Medizinische Labordiagnostika AG, Lübeck, Germany). In this methodology, sera are manually incubated on slides coated with cell substrate. ANA in sera, if present, reacts with antigens present in those cells. After washing, the slides were incubated with fluorescein isothiocyanate (FITC) conjugated antibodies that bind to the patient’s antibodies and can be visualised under a fluorescent microscope (Olympus BX51, Olympus, Hachioji, Tokyo, Japan). The ANA positivity threshold was set at a titre of ≥1/100. All analyses were performed at the Department of Laboratory Diagnostics, UHC Zagreb, and AC staining patterns were performed according to the International Consensus of Antinuclear Antibody Patterns (ICAP) guidelines with expert level reporting [41,44].

### 2.4. Statistical Analysis

Data were described overall and stratified by chronic uveitis status using gtsummary (v2.5.0) [46]. Categorical variables were reported as counts and percentages and were compared using Fisher’s exact test (nominal variables) or the Cochran–Armitage test for trend (ordinal variables). Continuous variables were reported as medians with interquartile range and compared using the Wilcoxon rank-sum test. AC pattern co-occurrence was presented with an UpSet-style plot using ggplot2 (v4.0.2) and cowplot (v1.2.0) [47,48,49]. To evaluate the potential of AC patterns for predicting JIA-U, a series of logistic regression models was fitted. All models used JIA-U as the dependent variable, whereas predictors differed by the inclusion of ANA status and specific AC patterns (AC-1 (diffuse according to previous nomenclature), AC-4 (fine granular), and AC-5 (spliceosome/nuclear matrix)) and their interactions. A “baseline model” was first defined by considering clinically relevant variables (sex, age at diagnosis, follow-up duration, and JIA subtype). These variables were further refined using stepwise selection in both directions, guided by the Akaike Information Criterion (AIC), to obtain a parsimonious adjustment set and avoid complex overfitting models. All subsequent models were constructed by adding ANA status (“referent” model) or AC pattern variables to the baseline model. Model comparisons were performed using likelihood ratio tests against the baseline and referent models. False discovery rate was controlled using the Benjamini–Hocberg method. The level of statistical significance was set to α = 0.05. Statistical analyses were conducted in R (v4.5.2) [50].

## 3. Results

Across the JIA cohort (N = 217), there were 139 (64%) female patients with a female to male ratio of 1.8:1. oJIA was the most common type of disease in 117 (54%) patients, followed by enthesitis-related arthritis (ERA) in 44 (20%) patients and pJIA in 33 (15%) patients. The cohort was divided into two subgroups: JIA-U and JIA without uveitis. In the subgroup of JIA-U patients (n = 56, 26%), there were 41 (73%) female patients with a female to male ratio of 2.7:1. JIA-U preceded JIA onset in 8 (14%) patients. The most common type of JIA was oJIA in 44 (79%), followed by pJIA in 6 (11%) patients. Patients with JIA-U were substantially younger at the time of JIA diagnosis, with a median age of 38 months (24, 72) compared with 99 months (36, 157) among those without JIA-U. The follow-up period was longer in patients with JIA-U compared to JIA without uveitis: 62 months (29, 70) vs. 40 months (20, 59), respectively (Table 1).

Inflammatory markers were broadly similar between the groups, with a median CRP of 2 mg/L (1, 5) in the JIA-U group versus 3 mg/L (1, 16) in the JIA without uveitis group, and a median ESR of 17 mm/h (10, 27) versus 17 mm/h (8, 35), respectively. Blood counts were comparable, with small differences in lymphocyte and eosinophil percentages. Ferritin levels were lower in the JIA-U group (35 μg/L (21, 49) vs. 47 μg/L (28, 74)). Serum proteins and immunoglobulin levels showed no major differences between the groups (Table 1).

In the JIA subgroup without uveitis (n = 161, 74%), stable disease remission was commonly achieved with methotrexate (MTX) in 63 (39%) and NSAIDs in 50 (31%) patients, whereas in the JIA-U subgroup, it was commonly achieved with biological DMARDs in 36 (64%) and MTX in 14 (25%) patients. (Table 1).

HEp-2 IFA (ANA) positivity was substantially higher among patients with JIA-U (48/56, 86%) compared to JIA patients without uveitis (83/161, 52%).

The AC-1 pattern was the most frequently observed pattern, present at any intensity in 94 of 217 children (43%). The pattern was markedly enriched among children with JIA-U, present in 39 of 56 (70%), compared with 55 of 161 (34%) in the JIA without uveitis group. High levels of AC-1-associated antibody semiquantitative titres (i.e., HEp-2 IFA staining intensity) (++/+++) were also more commonly detected in the JIA-U group (22/56, 39.3%) than in the JIA without uveitis group (19/161, 11.8%).

The AC-4 pattern was the second most prevalent, detected in 30 of 217 children (14%). AC-4 was more common in the JIA without uveitis group (28, 17.4%) compared to the JIA-U group (2/56, 3.6%).

Other AC patterns—including AC-5, AC-8 (no equivalent in previous nomenclature), AC-9 (no equivalent in previous nomenclature), AC-15 (actin-like), AC-16 (no equivalent in previous nomenclature), AC-19 (cytoplasmic homogeneous), AC-20 (cytoplasmic speckled), AC-22 (no equivalent in previous nomenclature), AC-23 (no equivalent in previous nomenclature), and AC-24 (centrioles)—were rare overall (each typically ≤3%), with very few cases in the JIA-U group.

**Figure 1:**
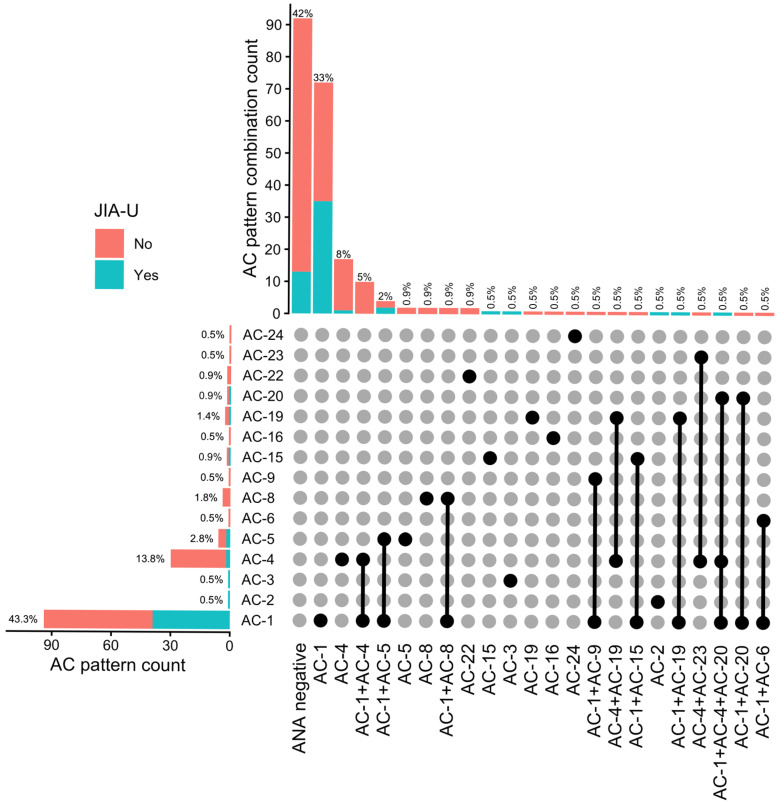
UpSet-style visualization of AC pattern combinations stratified by JIA-U. This figure shows how AC patterns occur individually and in combination. The top panel displays the frequency of each observed AC pattern combination (columns). The bars are stacked by JIA-U status (No/Yes). The labels above each bar indicate the proportion of the total cohort represented by that combination. The left panel shows the total frequency of each individual AC pattern (rows). Percentages indicate the proportion of participants in whom a given AC pattern was present. Because individuals may exhibit multiple AC patterns (or none), these percentages do not sum to 100%. The matrix panel (bottom right) links these two summaries. Each column corresponds to a specific AC combination shown in the top panel. Black dots indicate which AC patterns are included in that combination, and vertical lines connect patterns that co-occur. Columns (combinations) are ordered by decreasing frequency, so the most common combinations appear first from left to right. Rows (AC patterns) follow a fixed order based on AC nomenclature. Together, the figure allows the identification of the most frequent AC patterns and combinations, as well as their distribution across patients with and without JIA-U. Legend: JIA-U—JIA-associated uveitis, ANA—antinuclear antibody, AC—anti-cell.

Across all AC patterns, patients with JIA-U showed a concentrated predominance of AC-1, whereas JIA patients without uveitis demonstrated a broader distribution, including a moderate presence of AC-4 with minor contributions from other patterns (Table 2, Figure 1).

The HEp-2 IFA (ANA) status (referent) model demonstrated a substantially improved fit compared with the baseline model, with a deviance reduction of 7.4 (df = 1; *p* = 0.007; BH-adjusted *p* = 0.023), and had the most favourable information criteria among the evaluated models (AIC = 221.7; BIC = 248.7).

Models incorporating AC patterns (AC-1, AC-4, and their combinations) all improved fit relative to the baseline model. The AC-1 (χ^2^ = 10.6; df = 4; *p* = 0.031; BH *p* = 0.043) and AC-4 (χ^2^ = 13.0; df = 4; *p* = 0.005; BH *p* = 0.023) models showed significant improvement compared to the baseline model.

However, when compared with the HEp-2 IFA status base model, none of the AC pattern models achieved a significantly superior fit. AC-1, compared to the referent model, reduced the deviance by 3.3 (df = 3; *p* = 0.353; BH *p* = 0.503), AC-4 reduced the deviance by 5.6 (df = 3; *p* = 0.061; BH *p* = 0.231), and AC-5 showed poorer performance than the referent model, as reflected in negative deviance differences. The combined models AC-1 + AC-4 and AC-1 + AC-4 + AC-5 significantly improved the fit relative to the baseline but did not surpass the referent HEp-2 IFA status model (e.g., AC-1 + AC-4: χ^2^ = 18.1; df = 11; *p* = 0.080; BH *p* = 0.231).

Although models using AC patterns show lower deviance than the referent model, HEp-2 IFA (ANA) positivity alone remains the simplest independent predictor of JIA-U uveitis risk, yet its predictive performance is not significantly different from that of models based on AC patterns or their interactions (Table 3).

## 4. Discussion

To the best of our knowledge, this study is the first to investigate the association between specific AC patterns and semi-quantitative titres of associated specific antibodies, as defined by the ICAP nomenclature, exclusively in JIA-U patients compared to JIA patients without uveitis.

Because our JIA cohort is of moderate size, this study aligns most closely with the JIA studies by Küçükali et al., Saleh et al., and Papadopoulou et al. [6,38,51]. When examining the distribution of JIA subtypes, our cohort resembles European and North American studies, showing a female-to-male ratio of 1.8:1 and oJIA as the most common subtype, accounting for 45% of JIA patients without uveitis and 79% of JIA-U patients [1,9,10,14,38]. However, in our cohort, enthesitis-related arthritis (ERA) was the second most frequent subtype, occurring in 20% of all JIA patients instead of the expected 5–10%, and pJIA RF+ was identified in only 0.5% of all JIA patients instead of the 3–10% reported in most papers [1,6,9,10,18,38]. We mainly attribute these discrepancies to the size of our cohort rather than to specific characteristics of the Croatian population.

JIA-U was identified in 25.8% of all juvenile idiopathic arthritis (JIA) patients, with a female-to-male ratio of 2.7:1. Oligoarticular JIA (oJIA) was the most frequent disease subtype, and patients with JIA-U were substantially younger at diagnosis, with a median age of 38 months compared with 99 months for those without JIA-U. In 14% of JIA-U cases, uveitis preceded the onset of JIA. Our findings are consistent with the latest studies, except for the higher proportion of uveitis that precedes JIA, which usually occurs in up to 10% of all JIA-U patients [12,14,15,16,17,18,19]. We believe this result at least partially illustrates the exceptional collaboration between paediatric rheumatology and ophthalmology specialists at our centre, where each child with idiopathic uveitis is required to be monitored by both teams, enabling prompt diagnosis in cases of JIA-U.

In our study, no routine laboratory findings proved useful for distinguishing between JIA patients without uveitis and those with JIA-U, although some other studies have suggested that elevated ESR or leucocytosis at JIA onset may be predictive for JIA-U [12,14,27,52].

Among our JIA patients without uveitis, stable remission was most frequently achieved with methotrexate (MTX) (37.9%) and NSAIDs (31.7%). In contrast, for JIA-U patients, stable remission was most often achieved with biological DMARDs (66.1%) and MTX (28.6%). These results correspond with current treatment guidelines, confirm that JIA-U presents greater management challenges than JIA alone, and demonstrate the substantially broad availability of contemporary JIA therapies in our centre [14,15,30,31,32,53,54]. Analysis of the basic characteristics of our cohort did not reveal any data relevant for predicting the onset of JIA-U, as suggested by some studies [14,27,52] (Table 1 and Table 2).

Using a single determination approach and a threshold of ≥1/100, Hep-2IFA (ANA) positivity was observed in 60% of all JIA patients; 52% of those without uveitis, and 86% of those with JIA-U. Our prevalence results are higher than the usual overall prevalence of ANA positivity, which is reported in the literature as 30–50% of JIA patients [20,29,36,38]. A single ANA determination approach is preferred in most studies rather than requiring two instances because it yields similar consistency in chronic JIA-U prediction [6,8,20,22]. The prevalence of ANA positivity in our JIA-U patients aligns with the higher end of the reported range, spanning 14–98% across studies [20,22,28,36,38,39,55]. Apart from differences among JIA cohorts, the possible explanations for our results include a variety of thresholds used in different studies, ranging from 1/80 to 1/160 [6,10,20,22,29,35]. The significance of a positive ANA finding in up to 18% of healthy children and in up to 50% of patients with JIA has yet to be fully understood [6,8,15,20,22,29,36,37,38]. In healthy children, ANA is assumed to play a role in maintaining immune homeostasis, whereas its production in JIA patients is considered a genetically driven phenomenon associated with a specific subgroup of patients (i.e., early-onset, ANA positive) [3,8,16,22,28,36,38]. Positive ANA results are linked to a higher risk of developing chronic JIA-U, comorbid autoimmune diseases in JIA, and failure of DMARD therapy [3,6,8,9,20,22,35,36,38]. Conversely, ANA positivity does not increase the risk of developing JIA, is not useful for predicting the timing or severity of JIA-U and does not predict relapse of JIA when TNF inhibitors are withdrawn from patients in remission [8,14,29,36,38]. Furthermore, patients with oJIA who share the same ANA status can be categorised into subgroups characterised by a high titre of specific ANA autoantibodies, such as histone or chromatin, which are associated with increased JIA activity, and subgroups with low levels of these autoantibodies—comparable to healthy controls—and reduced disease activity [36]. Among patients with pJIA RF+, who are generally HLADRB1*08 negative, ANA positivity does not increase the risk of JIA-U [38]. All subsequent considerations raise the question of whether ANA positivity alone could serve as a marker in stratifying JIA subtypes.

The HEp-2 IFA detects dozens of autoantibodies against numerous intracellular antigens, providing substantially more information than the traditional ANA test, that is, the AC pattern (specific autoantibodies) and the level of specific autoantibodies (fluorescence intensity-semi-quantitative titre) [35,41,42,43,44]. According to some studies, it is expected that some AC patterns may be more predictive of JIA-associated comorbidities than others [22,35].

In our study, the AC-1 pattern was the most frequently observed pattern, present at any intensity in 43% of all JIA patients. The pattern was markedly enriched among children with JIA-U, present in 70% compared with 34% in the JIA without uveitis group. High levels of AC-1-associated antibody semi-quantitative titres (i.e., HEp-2 IFA staining intensity ++/+++) were also more common in the uveitis group (39%) than in the JIA without uveitis group (11.8%).

Regarding AC patterns in all JIA patients, our findings align with those of Baba et al. and Shin et al., who identified AC-1 as the most frequent pattern in 28.3% and 50% of JIA patients, respectively [29,56]. In relation to JIA-U, our findings most closely align with those of Koru et al., who examined and correlated AC patterns with quantitative ANA titres in 91 patients—12 with JIA-U, 21 with idiopathic uveitis, and 58 with JIA without uveitis. They reported an AC-1 pattern in 65.7% of JIA patients without uveitis and in all JIA-U patients [28]. Conversely, Sener et al. found the AC-1 pattern to be the second most frequent (following AC-4/5) in roughly one-quarter of all JIA patients and in about one fifth of JIA-U patients [22].

The association between the AC-1 pattern and nuclear antigens, i.e., chromatin, nucleosomes, and histones, suggests that specific antibodies targeting these antigens, particularly at high titres, play a role in the aetiopathogenesis of JIA-U [28,29,35,36,37,44]. JIA-U is characterised by significant B-cell involvement and local antibody production, which strongly depends on disease duration and subsequent time-dependent epitope spreading [16,17,21,22,25]. Among autoantibodies against nuclear antigens related to JIA-U, anti-histone antibodies were identified as a significant predictor of chronic JIA-U in several studies [14,15,28,29]. Arve-Butler et al. identified 17 JIA-U-specific autoantigens, of which five were nuclear autoantigens [57]. Heat-shock cognate 71-kDa proteins were also found to be potential nuclear antigens linked to JIA-U development [16].

In 64% of our JIA patients without uveitis, stable remission was achieved following early initiation of MTX alone or combined with bDMARDs, prompted by the heightened risk of an adverse disease course [11]. MTX therapy, particularly when initiated early, reduces the risk of JIA-U [51]. A further decrease in the risk of JIA-U was noted in JIA patients receiving TNFα inhibitors alone or in combination with MTX [12]. In our JIA-U group, uveitis was detected for the first time in 14 of 56 patients following the cessation of MTX, TNF-α inhibitors, or their combination, which had been ceased due to a prolonged and stable remission of the first JIA episode without uveitis. According to the literature, the risk of JIA-U development is increased up to 7 years from the onset of JIA [9]. All these factors can at least partially explain the lack of uveitis development in all AC-1-positive patients.

In our JIA cohort, the AC-4 pattern ranked second in frequency (14%) but occurred more often in JIA patients without uveitis (17.4%) than in JIA-U patients (3.6%). Our results are completely opposite to those reported by Sener et al., who used a similar methodology (at least one HEp-2 IFA (ANA test) with an ANA threshold titre ≥ 1/100) but reported the AC-4/5 pattern as the most common in all JIA patients (29.7%) and JIA-U patients (54.7%) [22].

AC-4 is the most common pattern observed in routine HEp-2 IFA testing [41]. It is frequently detected in both HEp-2 IFA-positive asymptomatic individuals and patients with various systemic autoimmune rheumatic diseases [41]. Thus, the identification of AC-4 in patients with JIA possibly indicates a partial overlap of the aetiopathogenetic mechanisms common to various autoimmune diseases [9,20,22,28]. Similarly, the rare detection of other AC patterns in our cohort may point to alternative triggers or pathogenetic mechanisms in JIA and JIA-U.

Our study is the first to use semi-quantitative titres (i.e., HEp-2 IFA staining intensity) of AC-associated antibodies, as suggested by ICAP [41,44]. High levels of AC-1 associated antibodies (++/+++) were more frequently observed in the JIA-U group (39%) compared to the JIA group without uveitis (11.8%). Our findings are partially consistent with the study conducted by Küçükali et al., which identified quantitative ANA titres ≥ 1/160 as having the strongest association with JIA-U [6]. In general, according to Vulsteke et al., the positive predictive value for different AID depends on the combination of AC pattern and the titre of specific antibodies [58].

Finally, our study did not demonstrate the superiority of AC patterns over traditional ANA determination in assessing the risk of JIA-U development. The protective effect of early DMARD therapy introduction on the occurrence of uveitis and changes in the JIA phenotype during the disease course partly explains our results [4,10,12,14,51]. Nevertheless, we believe that the determination of AC patterns and their utilisation in assessing the risk of JIA-U holds promise, which will be fully revealed in studies with longer follow-up and larger cohorts of JIA patients.

The strengths of our study include a well-defined Croatian cohort of JIA patients diagnosed according to ILAR criteria, and the use of AC patterns and semi-quantitative titres of specific autoantibodies in line with the latest ICAP recommendations. The limitations of our study include a relatively small cohort size and a short follow-up period, which may restrict the detection of associations between specific AC patterns and JIA subgroups, and an exclusive focus on uveitis presence without addressing its severity or complications.

## 5. Conclusions

Our study indicates that JIA-U is closely linked to AC-1 staining patterns and high semiquantitative titres of associated antibodies.

Although we have not proven the superiority of AC patterns over ANA positivity in the prediction of JIA-U, our results provide additional insights into the pathogenesis of this most serious extra-articular complication of JIA and underscore the significance of new PRINTO classification criteria for JIA, based on aetiopathogenesis.

The potential clinical relevance of AC patterns in children with JIA and JIA-U requires further multicentre studies involving larger cohorts and extended follow-up periods.

## Figures and Tables

**Table 1:** Baseline characteristics of the cohort stratified by JIA-U status and comparison of the characteristics between patients with and without uveitis.

Characteristic	JIA N = 217 ^1^	JIA Without Uveitis n = 161 ^1^	JIA-U n = 56 ^1^	*p*-Value ^2^
***Demographics***				
*Sex*				0.108
M	78 (36%)	63 (39%)	15 (27%)	
F	139 (64%)	98 (61%)	41 (73%)	
Age at JIA diagnosis (months)	72 (29, 147)	99 (36, 157)	38 (24, 72)	<0.001
Follow-up duration (months)	44 (22, 68)	40 (20, 59)	62 (29, 70)	0.010
***Clinical***				
*JIA subtype*				<0.001
Systemic	17 (7.8%)	16 (9.9%)	1 (1.8%)	
Oligoarticular	117 (54%)	73 (45%)	44 (79%)	
Polyarticular	33 (15%)	27 (17%)	6 (11%)	
Psoriatic JIA	6 (2.8%)	6 (3.7%)	0 (0%)	
Enthesitis-related arthritis	44 (20%)	39 (24%)	5 (8.9%)	
*Stable remission achieved on*				<0.001
NSAIDs	54 (25%)	50 (31%)	4 (7.1%)	
MTX	77 (35%)	63 (39%)	14 (25%)	
bDMARDs	80 (37%)	44 (27%)	36 (64%)	
tDMARDs	6 (2.8%)	4 (2.5%)	2 (3.6%)	
***Laboratory***				
ESR (mm/h)	17 (8, 32)	17 (8, 35)	17 (10, 27)	0.768
CRP (mg/L)	3 (1, 10)	3 (1, 16)	2 (1, 5)	0.018
Red blood cells (×10^12^/L)	4.58 (4.33, 4.82)	4.61 (4.33, 4.85)	4.53 (4.31, 4.77)	0.309
Haemoglobin (g/L)	123 (113, 132)	123 (113, 133)	120 (112, 126)	0.076
Haematocrit (%)	0.36 (0.34, 0.39)	0.36 (0.34, 0.39)	0.35 (0.33, 0.37)	0.094
White blood cells (×10^9^/L)	8.5 (6.8, 10.9)	8.5 (6.7, 11.0)	8.9 (7.3, 10.7)	0.714
Neutrophils (%)	53 (46, 62)	56 (46, 64)	50 (44, 56)	0.006
Lymphocytes (%)	36 (28, 43)	34 (26, 43)	39 (32, 45)	0.017
Monocytes (%)	7.10 (5.40, 9.10)	7.10 (5.40, 9.00)	7.35 (5.00, 9.20)	0.829
Eosinophils (%)	1.90 (1.00, 3.00)	1.60 (0.90, 2.90)	2.25 (1.00, 3.55)	0.038
Basophils (%)	0.40 (0.20, 0.70)	0.40 (0.20, 0.70)	0.50 (0.00, 0.85)	0.998
Platelets (×10^9^/L)	340 (283, 419)	325 (277, 410)	368 (303, 428)	0.028
Serum iron (μmol/L)	9.0 (6.0, 14.0)	9.0 (6.0, 14.0)	9.0 (6.0, 12.0)	0.756
Serum ferritin (μm/L)	42 (26, 65)	47 (28, 74)	35 (21, 49)	0.001
Total proteins (g/L)	72.0 (69.0, 76.0)	73.0 (69.0, 76.0)	71.0 (69.0, 74.0)	0.197
Albumin (g/L)	41.8 (39.5, 44.2)	42.0 (39.1, 44.4)	41.8 (40.0, 43.7)	0.922
Alpha-1 (g/L)	3.40 (2.90, 4.10)	3.40 (2.90, 4.20)	3.45 (2.80, 3.75)	0.300
Alpha-2 (g/L)	8.90 (7.50, 10.60)	8.80 (7.40, 10.60)	8.90 (7.90, 10.65)	0.516
Beta (g/L)	7.40 (6.80, 8.30)	7.60 (7.00, 8.50)	7.10 (6.60, 7.85)	0.003
Gamma (g/L)	10.6 (8.9, 13.0)	10.8 (8.9, 13.1)	10.5 (8.7, 12.3)	0.469
IgG (g/L)	11.5 (9.5, 13.9)	11.5 (9.7, 14.1)	10.9 (9.1, 13.3)	0.197
IgA (g/L)	1.11 (0.61, 1.66)	1.15 (0.70, 1.83)	0.88 (0.53, 1.43)	0.014
IgM (g/L)	1.16 (0.88, 1.56)	1.17 (0.86, 1.58)	1.15 (0.94, 1.41)	0.863

Legend: ^1^ n (%); Median (Q1, Q3), ^2^ Fisher’s exact test, Wilcoxon rank-sum test, JIA—juvenile idiopathic arthritis, JIA-U—JIA-associated uveitis, M—male, F—female, NSAIDs—non-steroidal anti-inflammatory drugs, MTX—methotrexate, bDMARDs—biological disease modifying anti-rheumatic drugs, tDMARDs—targeted DMARDs, ESR—erythrocyte sedimentation rate, CRP—C-reactive protein, Ig—immunoglobulin.

**Table 2:** Baseline characteristics of the cohort stratified by JIA-U status and comparison of the characteristics between patients with and without uveitis.

Characteristic	JIA N = 217 ^1^	JIA Without Uveitis n = 161 ^1^	JIA-U n = 56 ^1^	*p*-Value ^2^
HEp-2 IFA (ANA) positivity	131 (60%)	83 (52%)	48 (86%)	<0.001
AC-1 pattern semiquantitative titre				<0.001
−	123 (57%)	106 (66%)	17 (30%)	
−/+	6 (2.8%)	5 (3.1%)	1 (1.8%)	
+	47 (22%)	31 (19%)	16 (29%)	
++	30 (14%)	13 (8.1%)	17 (30%)	
+++	11 (5.1%)	6 (3.7%)	5 (8.9%)	
AC-4 pattern semiquantitative titre				0.023
−	187 (86%)	133 (83%)	54 (96%)	
−/+	11 (5.1%)	11 (6.8%)	0 (0%)	
+	15 (6.9%)	13 (8.1%)	2 (3.6%)	
++	4 (1.8%)	4 (2.5%)	0 (0%)	
+++	0 (0%)	0 (0%)	0 (0%)	
AC-5 pattern semiquantitative titre				0.965
−	211 (97%)	157 (98%)	54 (96%)	
−/+	1 (0.5%)	0 (0%)	1 (1.8%)	
+	4 (1.8%)	3 (1.9%)	1 (1.8%)	
++	1 (0.5%)	1 (0.6%)	0 (0%)	
+++	0 (0%)	0 (0%)	0 (0%)	

Legend: ^1^ n (%); Median (Q1, Q3), ^2^ Fisher’s exact test, Cochran–Armitage test for trend, JIA—juvenile idiopathic arthritis, JIA-U—JIA-associated uveitis, HEp-2 IFA—indirect immunofluorescence assay on HEp-2 cells, ANA—antinuclear antibody, AC—anti-cell, plus/minus signs—shorthands to indicate semiquantitative titre (from negative to highly positive).

**Table 3:** Comparison of multivariable logistic regression models for JIA-U. The baseline model included age at diagnosis (months; mean-centered), follow-up duration (months; mean-centered), and JIA subtype. The referent model added ANA positivity determined by HEp-2 IFA. Additional models evaluated AC pattern variables (AC-1, AC-4, AC-5), entered individually and in combinations, with pairwise interaction terms (e.g., AC-1 + AC-4, AC-1 + AC-5, AC-1 + AC-4 + AC-5). Model fit is summarized using AIC, BIC, log-likelihood, deviance, and residual degrees of freedom (Df). Likelihood ratio tests (LRTs) compare each model with the baseline model (“vs. baseline”) and, where applicable, with the referent model (“vs. referent”); *p*-values are reported as raw (*p_raw_*) and Benjamini–Hochberg (BH)-adjusted values (*p_adjusted_*).

						LRT vs. Baseline				LRT vs. Referent			
Model	AIC	BIC	log(Lik)	Deviance	Df	$\chi^{2}$	df	*p_raw_*	*p_adjusted_*	$\chi^{2}$	df	*p_raw_*	*p_adjusted_*
Baseline model													
baseline	227.1	250.7	−106.5	213.1	210	-	-	-	-	-	-	-	-
Referent model													
referent	221.7	248.7	−102.8	205.7	209	7.4	1	0.007	0.023	-	-	-	-
AC pattern models													
1	224.4	261.6	−101.2	202.4	206	10.6	4	0.031	0.043	3.3	3	0.353	0.503
4	220.1	253.9	−100.1	200.1	207	13.0	3	0.005	0.023	5.6	2	0.061	0.231
5	229.3	263.1	−104.6	209.3	207	3.8	3	0.283	0.283	−3.6	2	1.000	1.000
1 + 4	225.6	289.8	−93.8	187.6	198	25.4	12	0.013	0.030	18.1	11	0.080	0.231
1 + 5	228.6	279.3	−99.3	198.6	202	14.5	8	0.070	0.082	7.1	7	0.419	0.503
1 + 4 + 5	230.0	307.7	−92.0	184.0	194	29.1	16	0.023	0.041	21.7	15	0.116	0.231

Legend: AIC—Akaike information criterion, BIC—Bayesian information criterion, LRT—likelihood-ratio test, χ^2^—test statistics of the LRT, df—degrees of freedom of the LRT, AC—anti-cell.

## Data Availability

The data underlying this article will be shared on reasonable request by the corresponding author.

## Abbreviations

The following abbreviations are used in this manuscript:

AC	Anti-cell
JIA	Juvenile idiopathic arthritis
JIA-U	JIA-associated uveitis
HEp-2 IFA	HEp-2 immunofluorescence assay
PRINTO	Paediatric Rheumatology International Trials Organisation
ILAR	International League Against Rheumatism
HLA	Human leucocyte antigen
ANA	Antinuclear antibody
NSAIDs	Non-steroidal anti-inflammatory drugs
MARDs	Disease-modifying anti-rheumatic drugs
ESR	Erythrocyte sedimentation rate
CRP	C-reactive protein
MTX	Methotrexate
